# External stimulation induces switches between neural oscillations: an illustrative feedback model

**DOI:** 10.1186/1471-2202-12-S1-P156

**Published:** 2011-07-18

**Authors:** Axel Hutt

**Affiliations:** 1 Equipe Cortex, INRIA Nancy – Grand Est, Villers-les-Nancy, France

## 

Oscillatory activity in neural systems plays an important role in information processing. For instance, specific cognitive tasks evoke oscillations in certain frequency bands, which are characteristic for the corresponding experimental paradigm. Examples are the theta-rhythm (4-8Hz) in memory tasks or the gamma-rhythm (30-60Hz) in visual binding tasks. Moreover, changing the external activation of a neural system may lead to the activation of additional oscillations in specific frequency bands or may even yield a switch between specific frequency bands. In the last years, such a switch has been found experimentally in-vivo in some neural systems, such as in the mammalian olfactory bulb [1] and in the primary sensory area of weakly electric fish [2]. Both systems exhibit a feedback topology between two or more interacting populations and an external stimulation that originates from another brain area.

More detailed, the mammalian olfactory bulb is subject to the activation by the receptor cells, which are rhythmically activated by odours due to the breathing cycle. Applying an odour, the Local Field Potentials (LFP) exhibit high gamma-activity in the inhalation phase and switches to high beta-activity in the exhalationn phase. In weakly electric fish, the primary sensory area ELL receives input from electro-receptors on the skin of the fish. In the case of spatially uncorrelated noisy stimulation of the skin receptors, single neurons fire mainly in the beta-band, while a strong spatial input correlation switches on an additional spiking rythm in the gamma-band.

To explain such switches in frequency bands in feedback systems, the current work presents a neural population rate model of a rather general feedback system [3], that allows to derive mathematical conditions for the switch between frequency bands subject to the spatial correlation of an external input. The proposed model explains the switch between frequency bands as a selective activation of linear spatial oscillation modes. The centre frequency and the width of the bands and the properties of the spatial oscillation modes to be activated result from the intrinsic properties of the feedback system. Such properties are the synaptic time constants and delays between system components. Moreover, the spatial interactions in the feedback system determines the spatial frequency of the oscillation modes. The essential selection of the frequency band happens via the coupling of the spatially extended external input with the spatial modes. For illustration reasons, one may think of a guitar string, whose oscillation frequency depends on the manner how it is stricken.
